# Accelerator-Free Thermal Pose Estimation: Accuracy–Efficiency Trade-Offs on the Raspberry Pi 5

**DOI:** 10.3390/s26165188

**Published:** 2026-08-16

**Authors:** Gabriela Vdoviak, Tomyslav Sledevič, Dalius Matuzevičius

**Affiliations:** Department of Electronic Systems, Vilnius Gediminas Technical University, Saulėtekio Ave. 11, LT-10223 Vilnius, Lithuania; tomyslav.sledevic@vilniustech.lt (T.S.); dalius.matuzevicius@vilniustech.lt (D.M.)

**Keywords:** convolutional neural networks, raspberry Pi, keypoints detection, thermal images

## Abstract

Deploying human pose estimation on low-cost, accelerator-free hardware remains a key open question for smart home activity recognition, where affordability and wide availability often matter as much as raw inference speed. This work addresses that question by evaluating thermal-image pose estimation on a Raspberry Pi 5, a CPU-only single-board computer with no dedicated neural-network accelerator. Building on a previously collected single-person thermal dataset of 2500 images annotated with 17 body keypoints, YOLOv8-pose, YOLO11-pose, and YOLO26-pose models were trained across five model scales (*n*–*x*) at three input resolutions (640×512, 320×256, and 160×128 px) and deployed on the Raspberry Pi 5. Models were evaluated in native PyTorch, ONNX, and NCNN export formats, with per-image latency, power consumption, energy per frame, and pose mAP50–95 measured for each configuration. Pose accuracy scaled consistently with model size and input resolution, reaching up to 95.6% pose mAP50–95 for YOLO26-x at 640×512 px, with diminishing returns for the largest model scales. Export-format conversion to ONNX or NCNN preserved pose accuracy almost exactly relative to native PyTorch, with mean degradation below 0.6 percentage points, in contrast to the accuracy loss associated with numerical-precision reduction on GPU-accelerated platforms. NCNN consistently achieved the lowest per-image latency and energy consumption among the three formats, delivering up to 2.1–4.3× speed-up over PyTorch while keeping power draw up to 10 W across all configurations. The findings indicate that, unlike GPU-based edge deployment where precision reduction is the dominant efficiency driver, export-format optimization governs the latency–power–energy trade-off on CPU-only hardware, and that NCNN-based deployment on low-cost, widely available single-board computers offers a practical alternative for smart home thermal pose estimation.

## 1. Introduction

Information about posture, movement, and prolonged inactivity is essential for safety assessment and activity-aware services in domains such as smart-home monitoring, assisted living, rehabilitation, and healthcare [1,2,3,4,5]. However, systems designed for these settings must balance recognition performance with privacy, usability, cost, and local processing requirements. RGB cameras provide rich visual information, but they also capture identifiable appearance and private environmental details. Wearable and visual–inertial approaches can reduce dependence on fixed cameras, but they require the monitored person to carry sensors and may involve sensor-to-body calibration, which limits their suitability for passive long-term monitoring [6]. Consequently, privacy-preserving sensing and on-device inference have become central requirements for smart-home and human activity recognition systems [5,7,8,9,10].

Thermal imaging is suitable for privacy-preserving monitoring because it captures heat patterns rather than detailed visible appearance and remains usable in darkness or low-light conditions. Prior studies have applied thermal sensors to posture recognition, occupancy estimation, pedestrian analysis, and indoor human monitoring, demonstrating that thermal data can support human-state analysis without RGB imagery [11,12,13,14,15,16]. These applications require different forms of body-structure information, ranging from person-level detection and occupancy estimation to posture, activity, and keypoint-based analysis. For applications that depend on body configuration, human pose estimation provides a structured representation by localizing anatomical keypoints. However, in thermal imagery, keypoint localization remains challenging because reduced spatial resolution, weak anatomical texture, sensor noise, low contrast between adjacent body regions, occlusion, and residual heat artifacts can make joint positions difficult to estimate [17,18,19].

Beyond sensing modality and pose-estimation accuracy, practical monitoring systems are also limited by deployment requirements. The model must run continuously and within the computational and power limits of the target hardware. One deployment option is cloud-based inference, which can provide substantial computational resources, but introduces dependence on network connectivity, increases data-transfer requirements, and is less suitable for privacy-sensitive continuous monitoring [20]. Local inference avoids these limitations, but shifts the efficiency constraint to the deployed hardware and runtime environment. Workstation GPUs and embedded GPU platforms can accelerate pose-estimation models through parallel execution, TensorRT optimization, and reduced-precision inference, while external neural accelerators such as Edge TPU devices provide another route for low-power edge inference [21,22,23]. These approaches demonstrate that real-time thermal or pose-based analytics can be achieved on local or edge platforms, but their efficiency is often tied to specialized inference hardware, accelerator-specific optimization, additional hardware cost, and increased deployment complexity.

In contrast to accelerator-assisted deployment options, CPU-only deployment presents a different set of constraints. Without a GPU, TPU, or neural processing accelerator, inference performance depends more directly on model scale, input resolution, CPU operator implementation, memory behavior, and runtime format. Raspberry Pi-based studies have shown the feasibility of local thermal long-lie detection, pose-informed elderly safety monitoring, and smart-building occupancy analysis [24,25,26]. However, prior studies generally evaluate downstream monitoring tasks rather than isolating the pose-estimation stage across model families, model sizes, input resolutions, and export formats. Since runtime conversion can substantially affect CPU inference behavior, deployment format becomes part of the accuracy–efficiency trade-off rather than only an implementation detail, especially when comparing PyTorch, ONNX, and mobile-oriented inference frameworks such as NCNN [27]. A systematic evaluation of these factors is therefore needed to determine how thermal pose-estimation accuracy, latency, power consumption, and energy per frame change under CPU-only execution.

This study investigates accelerator-free thermal human pose estimation on the Raspberry Pi 5 edge device. It reuses a single-person thermal pose dataset captured with a Lynx L15 thermal monocular camera and annotated with bounding boxes and 17 anatomical keypoints, previously collected and used in our prior work evaluating embedded GPU deployment of thermal pose-estimation models on Jetson platforms [28]. Reusing the same annotated dataset and trained model families across both studies enables a direct, data-matched comparison between precision-reduction-based optimization on embedded GPUs and export-format-based optimization on CPU-only hardware. YOLOv8-pose, YOLO11-pose, and YOLO26-pose models were trained across five model scales and evaluated at three input resolutions. The trained models were then deployed on the Raspberry Pi 5 CPU using PyTorch, ONNX, and NCNN formats. The evaluation jointly considers keypoint accuracy, inference latency, power consumption, and energy per frame, enabling the accuracy–efficiency trade-offs of thermal YOLO-pose deployment to be characterized under a consistent CPU-only deployment setting. The main contributions of this work are as follows:

- A comparative evaluation of YOLOv8-pose, YOLO11-pose, and YOLO26-pose models for thermal human pose estimation across five model scales and three input resolutions.
- An accelerator-free Raspberry Pi 5 deployment study comparing PyTorch, ONNX, and NCNN execution formats on CPU-only hardware.
- A joint accuracy–efficiency analysis of thermal YOLO-pose models using keypoint accuracy, inference latency, power consumption, and energy per frame.
- Identification of practical model, resolution, and runtime-format trade-offs for privacy-preserving thermal pose estimation on low-cost CPU-based edge hardware.
- A transferable deployment principle, established through a dataset- and architecture-matched comparison with our companion embedded-GPU study [28]: on CPU-only edge hardware, the dominant efficiency factor is export-format and runtime graph optimization rather than numerical-precision reduction, and this shift holds consistently across three model generations, five model scales, and three input resolutions.

We emphasize that the contribution of this work is not a new pose-estimation architecture, training procedure, or compression technique, but a controlled empirical characterization that isolates export-format conversion as an independent optimization axis on CPU-only hardware, matched against the numerical-precision axis previously characterized on embedded GPUs using the identical dataset and model families [28]. This matched, two-study design is what allows us to state, as a generalizable principle rather than a platform-specific observation, that the dominant optimization mechanism shifts from precision to export format when accelerator hardware is absent. To our knowledge, no prior thermal pose-estimation study has performed this precision-versus-format comparison under a controlled, same-data, same-architecture protocol, which is the specific scientific contribution of the present work.

## 2. Related Works

Thermal human pose estimation combines privacy-preserving visual sensing with keypoint-based representation of body posture and movement. This combination is particularly relevant for smart-home monitoring, where continuous operation must be achieved without exposing detailed visual appearance and without relying on remote processing. Previous studies have addressed different aspects of this problem, including thermal-domain keypoint estimation, robustness under low-visibility conditions, model optimization for embedded hardware, and application-level deployment on low-cost edge devices. This section reviews these contributions in relation to the sensing, modeling, and deployment challenges addressed in this work.

### 2.1. Thermal-Domain and Low-Visibility Human Pose Estimation

Thermal human pose estimation aims to recover anatomical keypoints from infrared imagery, where body structure is represented through temperature differences rather than visible texture. This modality is suitable for privacy-preserving and low-light monitoring, but it introduces challenges such as reduced spatial resolution, thermal noise, weak contrast between adjacent body regions, occlusion, and limited annotated datasets. These limitations have motivated approaches based on RGB-to-thermal supervision transfer, lightweight model design, temporal modeling, and fusion with complementary sensing modalities.

Early thermal pose estimation studies commonly adapted pose estimation models originally developed for RGB images. Chen et al. [18] proposed ThermalPose, a bottom-up multi-person pose estimation framework trained using supervision transferred from paired visible-light images, showing that thermal pose estimation can remain functional in dark environments, although performance decreased under occlusion and crowding. Smith et al. [19] compared multiple top-down and bottom-up pose architectures on thermal images and found that top-down methods, particularly ViTPose, achieved higher accuracy, while bottom-up methods were less affected by the number of people in the scene. Kuzdeuov et al. [22] further expanded the available thermal pose data through OpenThermalPose2 and showed that modern YOLO-pose models can achieve high accuracy when trained on larger and more diverse thermal keypoint annotations. These studies indicate that thermal pose estimation is feasible, but remains strongly dependent on dataset size, annotation quality, and domain adaptation strategy.

Other works have focused on model design for infrared or degraded visual conditions. Zang et al. [29] proposed LMANet, a lightweight multi-stage attention network for single-person pose estimation in far-infrared images, combining a MobileNetV3-based backbone with channel and spatial attention to improve the accuracy–efficiency balance. Cormier et al. [30,31] investigated thermal pose estimation under low-light conditions and emphasized the need for thermal-domain training and robust evaluation under degraded visibility. Related low-visibility work by Luo et al. [32] combined low-light enhancement, YOLO-Pose, and temporal action recognition, showing that pose reliability depends not only on the pose estimator, but also on the sensing conditions and preprocessing pipeline. Lupión et al. [33] proposed a multi-view thermal framework for 3D human pose estimation in smart-home environments, using synchronized thermal cameras and visible-spectrum supervision during dataset construction. Zhu et al. [34] introduced a dual-channel cascaded network for 3D pose estimation and gait measurement from a single infrared thermal video, combining 2D pose estimation with Transformer-based depth regression. In later work, Zhu et al. [1] addressed in-bed health monitoring by estimating 2D pose from thermal images and fusing the result with aligned depth data to recover 3D joint positions. These studies show that thermal pose estimation can support clinically relevant monitoring tasks, but improved robustness often depends on additional sensing hardware, temporal modeling, or workstation-class GPU evaluation.

Thermal and infrared sensing have also been studied in related human-analysis tasks that do not estimate full anatomical skeletons. Guo et al. [12] introduced a benchmark for identity-preserved human posture detection in thermal images and showed that even coarse posture categories are affected by occlusion, low contrast, and ambiguous body shapes. Zhang et al. [13] compared RGB and low-cost thermal cameras for YOLO-based indoor occupancy detection, reporting competitive thermal-camera performance in privacy-sensitive settings but also errors caused by overlapping occupants, residual heat, and heat-emitting non-human objects. Multimodal datasets such as HDIA [8] and OctoNet [35] further demonstrate the growing role of thermal and infrared sensing in human activity understanding.

Collectively, prior studies confirm the value of thermal sensing for privacy-preserving monitoring, while showing that keypoint-level thermal pose estimation remains constrained by image quality, annotation availability, occlusion, and computational complexity.

### 2.2. Thermal Edge Analytics and Accelerator-Assisted Deployment

Beyond pose estimation accuracy, several studies have examined how these models can be deployed in practical monitoring systems. In these works, real-time operation is commonly achieved through lightweight architectures, reduced-precision inference, embedded GPUs, or dedicated neural accelerators.

One approach is to pair low-cost single-board computers with external inference accelerators. Lupión et al. [21] proposed THPoseLite, a thermal pose estimation system for smart-home monitoring based on a Raspberry Pi 4, a FLIR Lepton thermal camera, and a Coral Edge TPU accelerator. Their approach used an automatic thermal labeling pipeline based on BlazePose pseudo-labels and trained a MobileNetV2-based model to estimate body landmarks from low-resolution thermal images. The final system used 8-bit quantization and Edge TPU inference, reaching real-time operation with low power consumption.

Embedded GPU platforms provide another common route for efficient pose estimation model deployment. Kuzdeuov et al. [22] evaluated YOLOv8-pose and YOLO11-pose models trained on OpenThermalPose2 and deployed them on an NVIDIA Jetson AGX Orin using TensorRT with FP32, FP16, and INT8 precision. Their results showed that TensorRT optimization can substantially improve inference efficiency, with FP16 offering a favorable balance between speed and accuracy, while INT8 provided further acceleration at the cost of larger pose-accuracy degradation. Wang et al. [36] combined lightweight pose estimators with a compact LSTM-based temporal model for activity recognition, reporting real-time performance on workstation-class GPU and CPU hardware alongside reduced throughput of roughly 11 FPS on a Jetson Orin Nano in multi-person settings. In a rehabilitation context, Abromavičius et al. [37] evaluated a calibration-free, markerless RGB-D pose pipeline using single- and dual-camera configurations on a Jetson Orin Nano, achieving 29 Hz and 22 Hz, respectively, with the dual-camera setup reaching 15.4 ± 2.8 mm mean joint position error. These studies illustrate that embedded GPU platforms can support accurate, real-time pose-related analytics beyond thermal-imaging applications. Boldo et al. [38] proposed a real-time distributed multi-camera 3D human pose estimation platform in which edge nodes perform local pose inference and transmit synchronized keypoints to a centralized aggregation unit. Their work further illustrates that real-time pose deployment at the edge often depends on heterogeneous CPU–GPU hardware, synchronization mechanisms, and distributed system design.

A system-level thermal analytics framework has also been proposed for smart-home and eldercare applications. Zeng et al. [23] introduced ThermiKit, an LWIR analytics stack that integrates thermal object detection, pose estimation, tracking, and natural-language querying through a micro-MCP interface. The system uses RGB-to-thermal supervision transfer to train compact thermal models and evaluates low-cost LWIR sensors with different resolutions. Its results are relevant because they show that keypoint estimation is more sensitive than detection to reduced thermal resolution and sensor noise. However, ThermiKit evaluates an integrated perception and interaction stack rather than isolating pose-estimator deployment across model families, input resolutions, export formats, power consumption, and energy per frame.

Taken together, accelerator-assisted deployment studies demonstrate that real-time thermal pose or pose-based human-analysis systems can be achieved on edge platforms when dedicated inference hardware, embedded GPUs, quantization, or system-level optimization are available.

### 2.3. CPU-Based Edge Deployment

Low-cost CPU-based platforms are relevant for smart-home monitoring because they support local processing without requiring cloud transmission or dedicated inference hardware [39]. However, pose-estimation models are computationally demanding, and their practical use on Raspberry Pi-class devices depends on the interaction between model architecture, input resolution, runtime backend, and application-level processing pipeline. Existing studies on such platforms therefore provide useful deployment context, even when they focus on RGB sensing, activity recognition, or occupancy monitoring rather than thermal keypoint estimation.

Several works have explored Raspberry Pi-based systems for human monitoring in residential or healthcare settings. Analia et al. [24] proposed a Raspberry Pi 5-based thermal monitoring system for long-lie detection, showing the relevance of low-cost thermal sensing for privacy-preserving home and care applications. The model achieved 0.98 accuracy and the deployed system supported real-time monitoring of prolonged immobility over a 15-min observation period. Although the method used pose-derived thermal features rather than evaluating a full thermal pose estimator, it demonstrates that Raspberry Pi 5 hardware can support privacy-preserving, keypoint-informed thermal monitoring. Wang et al. [25] proposed an edge-oriented elderly safety system on a Raspberry Pi 4B, combining YOLOv7-tiny-Pose, tracking, identity verification, and a temporal convolutional network for activity recognition. Their system improved application-level performance through frame skipping, dynamic windows, and selective execution of computationally expensive modules, but the main evaluation focused on activity classification rather than pose-estimation accuracy, export formats, or energy-aware benchmarking.

Other Raspberry Pi studies have examined smart-home and building-monitoring tasks using lighter inference pipelines. Gad et al. [7] proposed a Raspberry Pi 5-based smart-home automation framework that used structured sensor and appliance data with gradient-boosting models for activity prediction, achieving low-latency on-device inference and strong activity-classification performance. Liu et al. [26] used Raspberry Pi 5 in a smart indoor occupancy detection system that combined optimized camera placement, YOLOv8-based person detection, cross-camera identity tracking, and semantic scene interpretation. Their results showed that multi-view de-duplication can improve occupant counting, while performance remains sensitive to occlusion and appearance changes. Similarly, Sykes [10] and related pose-based fall-detection studies show the value of skeleton representations for home safety monitoring, but they usually evaluate downstream classification performance rather than the computational trade-offs of the pose-estimation stage itself. Beyond classification accuracy, recent work has also begun treating the interpretability of such temporal classifiers as a first-class training objective, for example, explanation-guided regularization of dynamic neural networks for time-series activity recognition [40], which is complementary to the efficiency-focused evaluation carried out here.

Beyond application-level system design, CPU-based deployment is also affected by the software runtime used to execute the model. While accelerator-assisted studies often rely on TensorRT, TFLite with Edge TPU, or GPU-specific reduced-precision inference, accelerator-free Raspberry Pi deployment depends more strongly on CPU-oriented runtimes and operator implementations. In this setting, export format can become a major efficiency factor because the same trained model may exhibit different latency and energy behavior when executed through PyTorch, ONNX Runtime, or mobile-optimized frameworks such as NCNN [27].

Existing CPU-based edge studies demonstrate either pose-informed monitoring with RGB or thermal-derived features, thermal monitoring without full pose-estimator benchmarking, or smart-home inference with non-pose models. Therefore, the combined setting of thermal imagery, full-body pose estimation, Raspberry Pi CPU-only execution, and joint evaluation of accuracy, latency, power consumption, and energy per frame remains underexplored.

More specifically, the literature reviewed above reveals two largely disjoint optimization paradigms for efficient pose-estimation deployment: numerical-precision reduction (FP16/INT8) on GPU- or NPU-class accelerators [22], and, separately, ad hoc CPU-oriented system engineering (frame skipping, selective execution) on accelerator-free boards [25]. No existing study directly contrasts these two paradigms under matched data, model families, and evaluation protocol. Because our companion GPU study [28] already characterizes the precision-reduction paradigm on Jetson platforms using the identical thermal dataset and the same YOLOv8- and YOLO11-pose families, the present work is positioned to supply the missing CPU-side counterpart, and thereby to test, for the first time under controlled conditions, whether the accuracy–efficiency factor that dominates on GPU-class hardware (precision) still dominates when that hardware is absent. The transferable insight this comparison yields, that it does not, and that export-format and runtime optimization instead governs the trade-off on CPU-only hardware, is a principle we expect to generalize beyond the specific models and dataset used here to other lightweight, single-stage pose architectures with mature CPU export tooling (Section 4.3).

## 3. Materials and Methods

**Figure 1:**
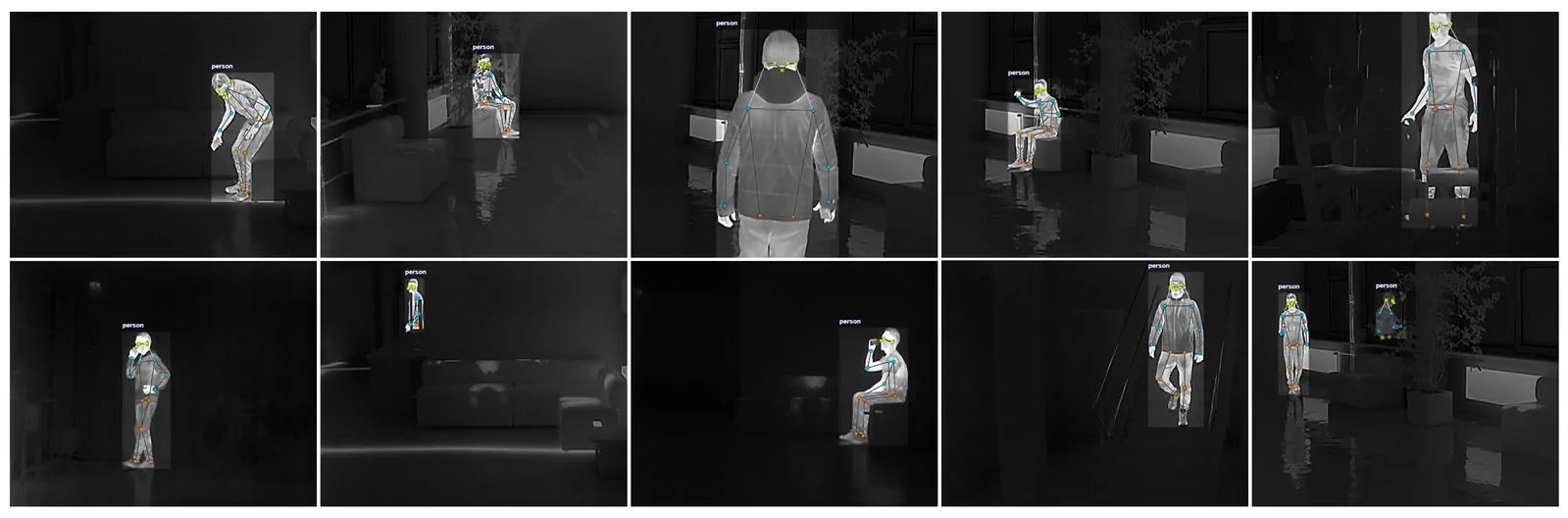
Example thermal images from the dataset with 17 annotated human pose keypoints.

A single-person thermal image dataset was collected to assess keypoint detection accuracy under the proposed deployment scenario. Thermal video was captured with a Lynx L15 thermal monocular at a spatial resolution of 720×576 px and a frame rate of 25 fps, and stored as MP4 sequences. To limit redundancy between temporally adjacent frames, every fifth frame was sampled from the recorded sequences for annotation. The resulting set of 2500 frames was manually labeled with a single-person bounding box and 17 body keypoints (Figure 1), in accordance with the standard human pose estimation annotation protocol. This dataset, and the training splits derived from it, are the same as those used in our previously published study of embedded GPU deployment on Jetson platforms [28], in which the same model families (YOLOv8-pose, YOLO11-pose) were evaluated under TensorRT precision reduction (FP32, FP16, INT8) on the Orin Nano and AGX Orin. The present study extends that investigation to the complementary, accelerator-free setting: identical model families and training data are here re-evaluated for CPU-only deployment on the Raspberry Pi 5, isolating export-format optimization (PyTorch, ONNX, NCNN) as the CPU-side counterpart to the GPU-side precision-reduction strategies examined previously, and additionally extending the model family coverage to YOLO26-pose.

The data were collected from a single, 36-year-old male participant of average build. To introduce variability in thermal silhouette, self-occlusion, and body-heat distribution, three clothing conditions were used during recording: a T-shirt, a sweater, and a jacket worn with a hat. Recordings were performed across eight different indoor rooms at an ambient temperature of approximately 21 °C, introducing variation in background clutter, furniture layout, and thermally reflective surfaces while keeping the participant and camera model fixed.

Pose estimation models from three successive YOLO-pose generations, YOLOv8, YOLOv11, and YOLOv26, were trained on the thermal dataset described above. Training was carried out on an NVIDIA GeForce RTX 4080 Super GPU with 16 GB of VRAM, using Ultralytics v8.3.80, Python 3.12.9, PyTorch 2.5.1, and CUDA 12.6. For each model family, training was repeated at three input resolutions, 640×512 px, 320×256 px, and 160×128 px, so that the effect of resolution on downstream edge-device performance could be isolated from the choice of architecture.

The three input resolutions were not selected arbitrarily, but follow a systematic dyadic downsampling scheme anchored to the native sensor output. The Lynx L15 thermal camera produces frames at 720×576 px, corresponding to a 5:4 aspect ratio. The highest evaluated resolution, 640×512 px, preserves this 5:4 aspect ratio while being the closest size divisible by 32, the maximum stride of the YOLO-pose backbones, thereby avoiding the padding or cropping artifacts that a non-multiple-of-32 input size would introduce. The two lower resolutions, 320×256 px and 160×128 px, are obtained by halving and quartering each spatial dimension of the base resolution, respectively (a 4× and 16× reduction in pixel count), while preserving the same 5:4 aspect ratio at every step. This dyadic ladder enables a controlled evaluation of the accuracy–efficiency trade-off across resolution, isolating the effect of spatial downsampling from confounds such as aspect-ratio distortion or backbone-incompatible input sizes, and spans a practically relevant range from near-native sensor resolution down to a resolution suited for maximum-throughput, low-power operation on the Raspberry Pi 5.

The annotated dataset was split into 80% for training and 20% for validation and testing, with the same partition reused across all model variants and resolutions to keep the comparison consistent. Critically, this split was performed at the recording-session level rather than at the frame level. The 2500 annotated frames originate from 23 separate MP4 recording sessions; each session was assigned in its entirety to either the training set (14 sessions, yielding 2000 frames) or the validation/test set (9 sessions, yielding 500 frames). Because temporally adjacent frames were sampled from the same continuous recording (every fifth frame, as described above), session-level assignment guarantees that no recording session, and therefore no temporally adjacent or near-duplicate frame pair, appears in both the training and test partitions. This separation can be independently verified from the publicly released dataset (see Data Availability Statement), in which each annotated image filename encodes its source recording session. A fixed random seed was applied throughout, controlling data shuffling, weight initialization, and the stochastic components of augmentation. During training, images were augmented with random translation of up to ±10% of the image width, scaling with a gain of ±0.5, and horizontal flipping applied with probability 0.5; mosaic augmentation was switched off for the final 10 epochs to stabilize convergence before completion.

All models were optimized with AdamW, using a learning rate of $1\times 10^{-3}$, a momentum coefficient of 0.9, and weight decay regularization. Training was allowed to run for up to 1000 epochs, with checkpoints saved every 100 epochs and early stopping triggered after 100 epochs without validation improvement, so as to avoid overfitting and unnecessary GPU time. Batch size was adapted automatically between 4 and 96 according to model size and input resolution to make full use of the available GPU memory; in practice, the validation loss of most configurations plateaued between epochs 200 and 400.

Prior to deployment, each trained PyTorch checkpoint was exported to ONNX and NCNN format using the Ultralytics export API (Ultralytics 8.4.23), with all export arguments specified explicitly rather than left at their library defaults. For each of the three training resolutions, the imgsz argument was set to the matching (height, width) pair (512,640), (256,320), and (128,160) rather than a single square value, so that the exported graph matches the aspect ratio and resolution used during training. Dynamic input shapes were disabled (dynamic = False), so that every exported model corresponds to a single fixed input resolution matching its PyTorch checkpoint. For ONNX exports, graph simplification via onnxslim was enabled (simplify = True, the library default), and the ONNX opset version used for each export was recorded directly from the exported graph (via onnx.load(...).opset_import) and is reported in Table 1. No numerical-precision reduction was requested during export (half/int8 left unset), so all ONNX and NCNN models are FP32, isolating export-format conversion as the only optimization axis distinguishing the three formats evaluated in this study. NCNN export accepts only imgsz, quantize, batch, and device as arguments (opset/simplify apply to the ONNX export path only) and used the same per-resolution imgsz values as above. End-to-end export was left at each model family’s library default: YOLO26-pose, which supports NMS-free end-to-end export, therefore used NMS-free postprocessing, while YOLOv8-pose and YOLO11-pose, which do not support end-to-end export, retained standard NMS-based postprocessing. We note this explicitly because Table 2 reports per-image time inclusive of postprocessing, and the difference in postprocessing pipeline between YOLO26 and the earlier two families is an architectural property of the models rather than a confound introduced by the benchmarking protocol.

**Figure 2:**
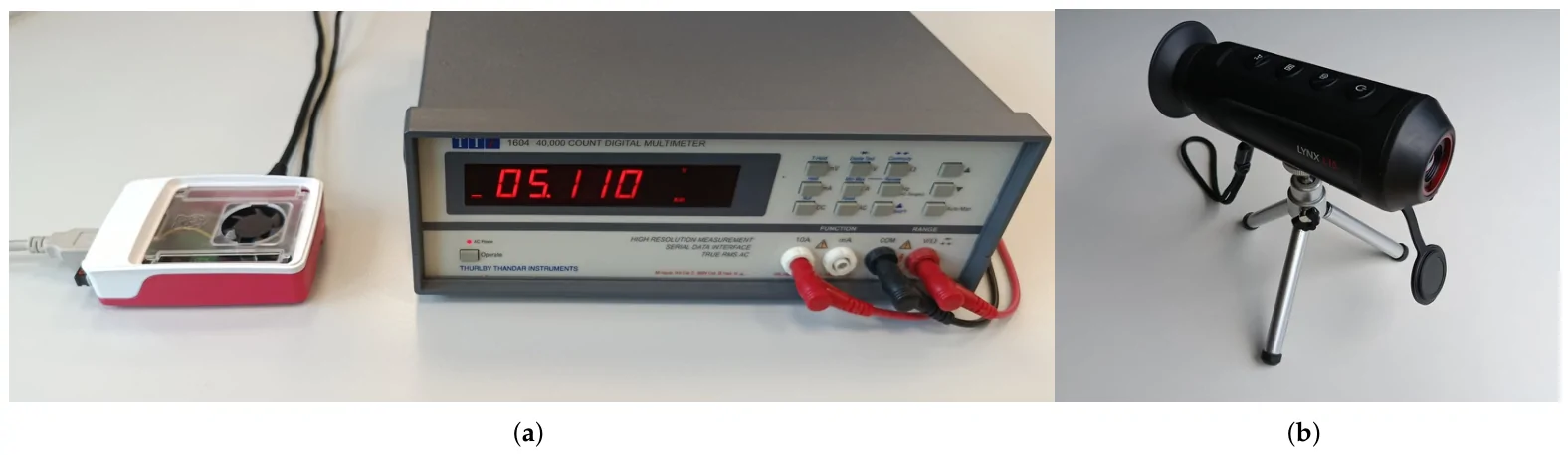
Raspberry Pi 5 deployment and measurement setup: single-board computer connected to a digital multimeter for power logging (**a**), and the Lynx L15 thermal monocular used for image acquisition (**b**).

Trained models were deployed and benchmarked on a Raspberry Pi 5 single-board computer, a CPU-centric platform built around the Broadcom BCM2712 system-on-chip with a quad-core Arm Cortex-A76 processor and 8 GB of LPDDR4X RAM, running 64-bit Raspberry Pi OS (Figure 2). The deployment software stack comprised Debian GNU/Linux 13 (“trixie”), Linux kernel 6.12.75 + rpt-rpi-2712, Python 3.13.5, PyTorch 2.10.0 (CPU build), ONNX Runtime 1.24.4, OpenCV 4.13.0, Ultralytics 8.4.23, and NumPy 2.4.3. Training used a separate environment (Ultralytics v8.3.80, Python 3.12.9, PyTorch 2.5.1, CUDA 12.6; see above), so the two software stacks differ deliberately between the GPU-based training stage and the CPU-only deployment stage evaluated here. Unlike GPU- or NPU-equipped edge boards, the Raspberry Pi 5 has no dedicated neural-network accelerator, so all inference in this study relies exclusively on the host CPU; this choice was deliberate, as it isolates the accuracy–latency–power trade-offs achievable on the kind of low-cost, widely available hardware most likely to be adopted in real residential deployments.

Electrical power drawn by the Raspberry Pi 5 during inference was measured with a TTi 1604 digital multimeter, which records current and voltage and streams the readings over a serial connection via a COM-to-USB converter to the same Raspberry Pi 5 under test. The TTi 1604 samples continuously at its fixed reading rate of 2.5 readings/s, with a manufacturer-specified basic accuracy of 0.08% of reading ±4 digits for DC voltage and 0.1–1.0% of reading ±4 digits for DC current (range-dependent), valid over the instrument’s calibrated 19–25 °C ambient temperature range, which matched the laboratory conditions under which all measurements were performed. For each of the 135 experimental configurations (three model families × five scales × three export formats × three resolutions), power was logged continuously while the model performed inference over the 500 images of the held-out validation split, and the value reported in Table 1 is the arithmetic mean of these per-image readings. Each configuration was executed as a single sequential run rather than repeated independently; run-to-run repeatability and the effect of sustained continuous operation on thermal throttling were not evaluated in the present study. On the device, a custom Python script logs three categories of measurements for each experimental configuration: instantaneous power readings sampled continuously from the multimeter, per-frame inference latency recorded while the model runs in YOLO prediction (inference) mode, and pose estimation accuracy obtained by separately running the model in validation mode against the held-out test split. All measurements are time-stamped and written to per-run CSV files for subsequent offline analysis.

To systematically characterize the accuracy–efficiency trade-off on the Raspberry Pi 5, the custom script drives an automated sweep over the full combination of experimental factors: the three YOLO-pose generations (YOLOv8, YOLOv11, YOLOv26), each at five model scales (nano, small, medium, large, and extra-large), exported to three inference formats (native PyTorch, ONNX, and NCNN), and evaluated at the three input resolutions used during training (640×512, 320×256, and 160×128 px). Each combination is executed as an independent, sequential run, with the multimeter continuously logging power draw alongside the script’s own logging of inference latency and validation accuracy, yielding a unified dataset for comparing model family, scale, export format, and resolution under identical hardware conditions. To ensure a fair, like-for-like comparison across export formats and to make full use of the Raspberry Pi 5’s four Arm Cortex-A76 cores, the CPU thread count available to each inference backend was fixed explicitly to four threads for every run (matching the number of physical cores), applied consistently to the PyTorch (torch.set_num_threads), ONNX Runtime (intra-op thread count), and NCNN (thread-count parameter) backends. This fixed, matched thread budget is applied uniformly across all 135 experimental configurations reported in Table 2.

The Raspberry Pi 5 CPU frequency governor was left at the Raspberry Pi OS default, ondemand, for the duration of all experiments, and the board relied exclusively on its integrated on-board fan for cooling, with no active external cooling. All measurements were performed at a laboratory ambient temperature of approximately 21 °C, consistent with the temperature range under which the training data were collected. No dedicated warm-up phase preceded logging for each configuration; the reported per-image latency statistics are computed over all 500 images of the validation run for that configuration. For each of the 135 configurations, in addition to the mean per-image latency reported in Table 2, the on-device inference script logged the per-run standard deviation of per-image latency, together with the mean and maximum on-chip CPU temperature and a binary thermal-throttling flag, both queried via vcgencmd (measure_temp and get_throttled, respectively) at the same cadence as the latency log. Power was measured concurrently and independently by the TTi 1604 digital multimeter described above, from which the per-run mean and standard deviation of power were computed over the same run time window. These per-configuration statistics, together with the raw per-image logs, are released as a supplementary CSV alongside the dataset described in the Data Availability Statement.

## 4. Results

This section presents the runtime and accuracy results of deploying YOLOv8-pose, YOLO11-pose, and YOLO26-pose models on the Raspberry Pi 5. The results include per-image inference latency, power consumption, and energy consumption per frame, measured across three export formats (native PyTorch, ONNX, and NCNN), five model scales (*n*–*x*), and three input resolutions. Pose estimation accuracy is reported using pose mAP50–95.

### 4.1. Inference Latency, Power Consumption, and Energy Efficiency

Table 2 reports the per-image time, power consumption, energy per frame, and pose mAP50–95 for all three YOLO-pose families across the three export formats and three input resolutions. Across most model families, scales, and resolutions, per-image latency decreases when moving from native PyTorch execution to ONNX, and decreases further and more consistently when moving to NCNN. The exception occurs for the largest model scales at the highest input resolution, where ONNX execution provides little to no latency benefit over native PyTorch, and can even be marginally slower (e.g., YOLOv8-x at 640 × 512 px: 2712 ms under PyTorch versus 2976 ms under ONNX). At 640×512 px, YOLOv8-pose latency is reduced from 209–2712 ms (PyTorch) to 172–2976 ms (ONNX) and 83–1007 ms (NCNN) across the *n*–*x* scales, corresponding to NCNN speed-ups of approximately 2.5–2.8× relative to PyTorch. A comparable trend is observed for YOLO11-pose and YOLO26-pose, where the largest (*x*) variants require 2876–2916 ms under PyTorch but only 1350–1370 ms under NCNN at the same resolution. Latency scales consistently with both model size and input resolution: reducing the resolution to 160×128 px lowers the NCNN latency of the nano variants to 8–9 ms, enabling frame rates well above real-time (over 100 FPS) irrespective of model family.

Power consumption follows a different pattern than latency. With all three formats run under the same fixed four-thread CPU budget, native PyTorch and NCNN execution draw comparable power, and are generally the two lowest-power formats, typically ranging between 6.4 W and 10.5 W; NCNN draws power at or slightly below the PyTorch level in nearly every configuration (ratio ≈0.83–1.01× relative to PyTorch), reflecting the efficiency of its ARM-optimized convolution kernels rather than reduced CPU utilization. ONNX Runtime execution, in contrast, consistently draws the highest power of the three formats, reaching up to 11.4 W for the largest models at 640 × 512 px, consistent with more aggressive multi-threaded CPU utilization; however, this higher power draw is not matched by a proportionally shorter execution time, and for the largest models at the highest resolution ONNX latency can equal or slightly exceed that of native PyTorch. As a result, NCNN, which combines the shortest execution time with power draw at or below the PyTorch baseline, consistently achieves the lowest energy consumption per frame among the three formats. For example, YOLOv8-x at 640 × 512 px requires 28.42 J (PyTorch), 33.84 J (ONNX), and only 9.68 J (NCNN) per frame, a ∼2.9-fold reduction in energy relative to PyTorch and a ∼3.5-fold reduction relative to ONNX. This pattern holds consistently across model families, scales, and resolutions in Table 2; only for a small number of the largest model configurations at 640 × 512 px does native PyTorch draw marginally less power than NCNN (e.g., YOLO26-l and YOLO26-x), in which cases NCNN’s energy advantage derives entirely from its shorter execution time rather than from lower power draw.

Pose accuracy (pose mAP50–95) increases with both model scale and input resolution, following the trend expected for keypoint detection under coarser spatial sampling. Across the three model families, pose mAP50–95 at 640×512 px ranges from 89.8% (YOLO11-n) to 95.6% (YOLO26-x), whereas at 160×128 px it drops to 73.8–89.3% over the same range of scales. YOLO26-pose almost consistently achieves the highest accuracy among the three families at a given scale and resolution, for instance, reaching 95.2–95.6% pose mAP50–95 for the *l* and *x* variants at 640×512 px, compared with 93.0–94.2% for YOLOv8-pose and YOLO11-pose in the same configurations.

### 4.2. Measurement Variability and Thermal Stability

Table 3 summarizes, aggregated by export format and input resolution across the five model scales and three model families (15 configurations per cell, 135 in total), the within-run coefficient of variation (CV, standard deviation divided by mean, expressed as a percentage) of per-image latency (logged by the on-device inference script) and instantaneous power (logged concurrently by the TTi 1604 digital multimeter), together with the mean and maximum on-chip CPU temperature and the number of configurations in which the Raspberry Pi 5’s thermal-throttling flag was raised at any point during the run.

Within-run dispersion is low across the great majority of configurations: averaged over all 135 runs, the latency CV is 2.3% (range 0.1–12.7%) and the power CV is 8.0% (range 2.3–10.9%). The handful of configurations with the highest latency CV are all among the shortest-duration runs, where per-image time is only a few tens of milliseconds and a small number of OS-scheduling interruptions therefore translate into a larger relative deviation despite a modest absolute one; for example, the ONNX YOLOv8-n configuration at 160 × 128 px has a mean per-image time of only 15.7 ms with a standard deviation of 2.0 ms (CV = 12.7%), and the NCNN YOLOv8-n configuration at 320 × 256 px shows a similar pattern (mean 19.8 ms, SD 2.4 ms, CV = 12.1%). In absolute terms, these deviations are on the order of 1–4 ms and do not materially affect the throughput or energy conclusions drawn from the mean values in Table 2. Power CV is more uniform across configurations, reflecting continuous fluctuation in CPU utilization during inference rather than measurement instability. Mean CPU temperature remained at or below 70.0 °C for every format/resolution cell, and the highest instantaneous temperature recorded across all 135 runs was 77.4 °C (ONNX, YOLOv8-m, 640 × 512 px). The firmware thermal-throttling flag was not raised in any of the 135 configurations. Under the fixed four-thread, ondemand-governor, fan-cooled, 21 °C ambient configuration used throughout this study, we therefore find no evidence that thermal throttling affected the latency or power figures reported in Table 1. We note that this analysis characterizes within-run measurement precision and thermal state; it does not substitute for independent between-run repetition, which would additionally capture run-to-run variability such as scheduling jitter or thermal drift between separate executions, and which remains a limitation of the present protocol (see Section 5).

### 4.3. Accuracy Preservation Across Export Formats

Table 4 summarizes the mean and standard deviation of the pose mAP50–95 degradation of ONNX and NCNN relative to native PyTorch, averaged over the five model scales (*n*–*x*) of YOLOv8-pose, YOLO11-pose, and YOLO26-pose at each input resolution. At 640×512 px, ONNX and NCNN show a small mean accuracy drop of −0.54±0.31 pp and −0.52±0.28 pp, respectively. This degradation narrows at 320×256 px to −0.2±0.13 pp for both formats, and becomes negligible, or even marginally positive, at 160×128 px (0.04±0.31 pp for ONNX and 0.02±0.24 pp for NCNN). These results indicate that format conversion to ONNX or NCNN on the Raspberry Pi 5 preserves pose accuracy almost exactly, with sub-percentage-point deviations and low variance across model scales and architectures. Consequently, the accuracy differences observed in Table 2 are governed primarily by model scale and input resolution rather than by the choice of export format.

**Figure 3:**
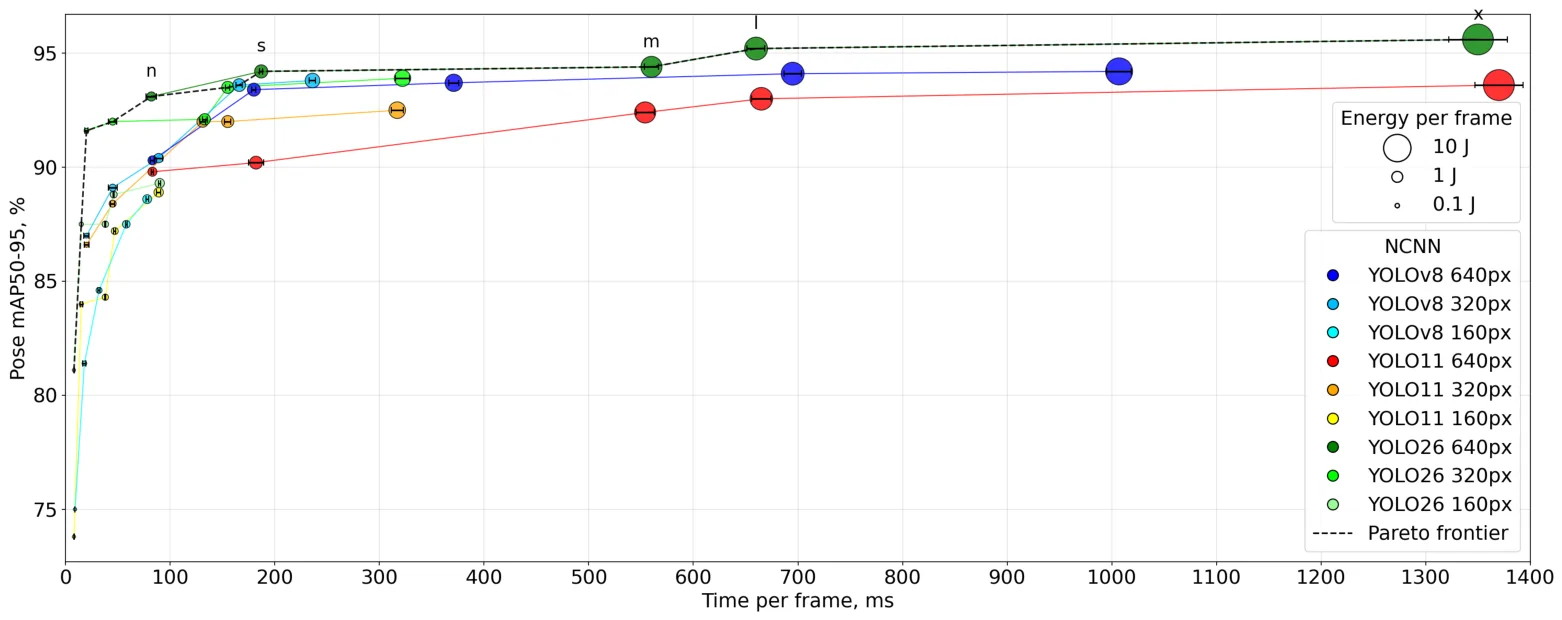
Accuracy–latency–energy trade-off for the NCNN format: pose mAP50–95 versus per-image time across all three YOLO-pose families, five model scales, and three input resolutions on the Raspberry Pi 5. Marker size denotes energy consumption per frame.

**Figure 4:**
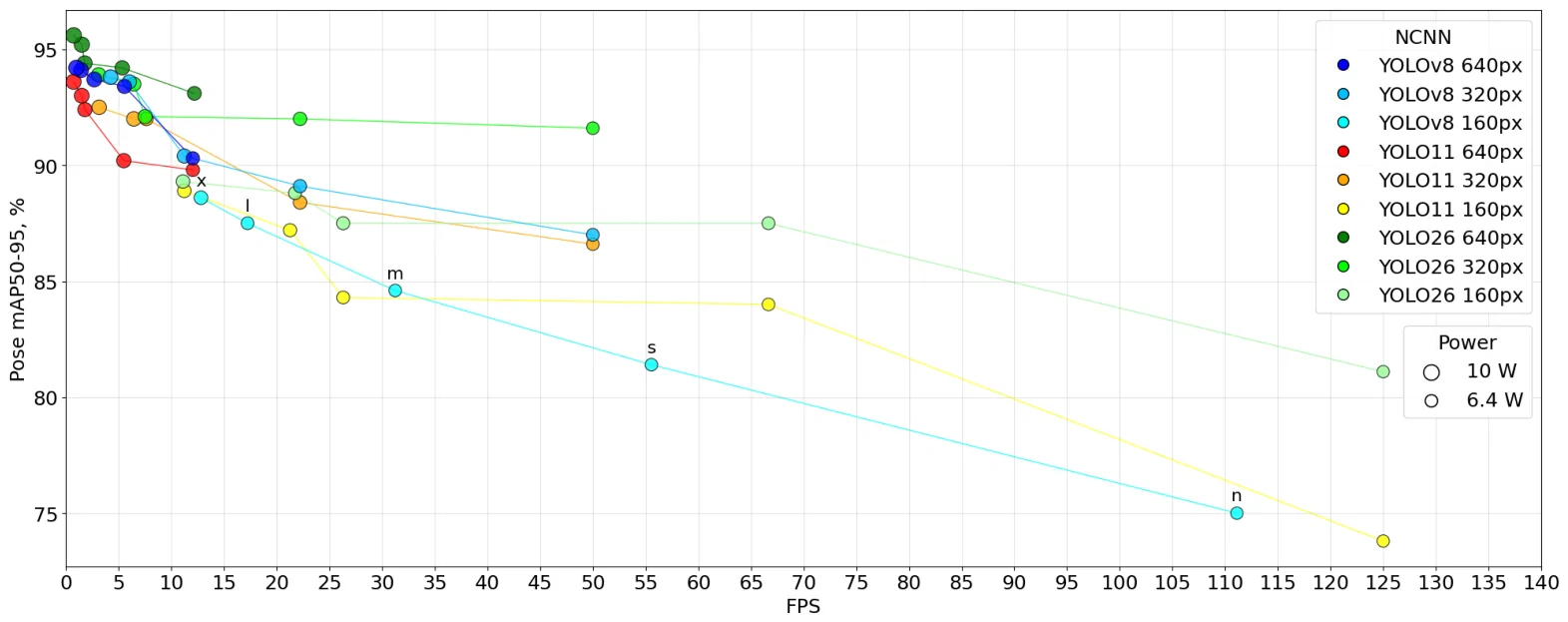
Accuracy–throughput–power trade-off for the NCNN format: pose mAP50–95 versus frame rate (FPS) across all three YOLO-pose families, five model scales, and three input resolutions on the Raspberry Pi 5. Marker size denotes power consumption.

Given that ONNX and NCNN preserve pose accuracy almost identically relative to PyTorch (Table 4), the choice of export format for deployment is therefore governed by runtime efficiency rather than accuracy loss. As shown in Table 2, NCNN consistently achieves the lowest per-image latency and the lowest energy consumption per frame among the three formats, while operating at power levels below those of ONNX, at comparable pose mAP50–95. Because NCNN thus dominates ONNX and PyTorch in both speed and energy efficiency without sacrificing accuracy, the remaining analysis of the accuracy–latency–power trade-off (Figure 3 and Figure 4) focuses exclusively on the NCNN format, which represents the most favorable operating point observed for deployment on the Raspberry Pi 5 under the single-subject, single-sensor conditions evaluated in this study.

Figure 3 illustrates the joint trade-off between pose mAP50–95, per-image time, and energy per frame for the NCNN format across all three model families, scales, and resolutions. Configurations at 160×128 px cluster at low latency, generally below 100 ms/frame, with pose mAP50–95 confined to approximately 74–89% and energy per frame below 0.1 J, marked by the smallest bubbles in the figure. Increasing the resolution to 320×256 px shifts the operating points to 20–322 ms/frame and 87–94% pose mAP50–95, while 640×512 px configurations extend the latency range up to approximately 1350–1370 ms/frame for the largest models, with the highest accuracies observed, up to 95.6% (YOLO26-x), and energy per frame reaching approximately 13 J for the largest, highest-resolution configurations. Across all three resolutions, the accuracy gain from the largest (*x*) relative to mid-sized (*m*) variants is comparatively small, whereas the increase in latency and energy is substantial, indicating diminishing accuracy returns for the largest model scales.

To make the accuracy–latency trade-off surface explicit, Table 5 lists the Pareto-optimal configurations among the 45 evaluated NCNN configurations, i.e., configurations for which no other evaluated configuration achieves both lower latency and equal-or-higher pose mAP50–95. Eleven of the 45 configurations are Pareto-optimal, and all but one (YOLOv8-l at 320 × 256 px) are YOLO26-pose, indicating that the newer architecture generation dominates the other two families across almost the entire accuracy range at matched latency. In Figure 3, the Pareto-optimal configurations listed in Table 5 are connected with a dashed line to visually separate dominant operating points from dominated ones. Regarding measurement precision, Table 3 reports the within-run coefficient of variation of per-image latency and power for the same 45 NCNN configurations; latency CV ranges from 0.8% to 12.1% (the highest values confined to the shortest-duration nano configurations discussed in Section 4.2) and power CV ranges from 2.3% to 10.9%, both small relative to the range of values spanned by the plotted operating points.

Figure 4 presents the corresponding trade-off between pose mAP50–95, frame rate (FPS), and power consumption for the NCNN format. The smallest models at the lowest resolution achieve the highest throughput, exceeding 100 FPS for the nano variants at 160×128 px, at the lowest power levels (approximately 6.4 W) and the lowest accuracy (74–81%). Conversely, the largest (*x*) models at 640 × 512 px operate at well below 1 FPS (NCNN latency of 1007–1370 ms/frame) while drawing power up to 10.0 W and achieving pose mAP50–95 up to 95.6%. Between these extremes, mid-sized models and intermediate resolutions occupy a continuous accuracy–throughput–power surface, confirming that, on the CPU-only Raspberry Pi 5 platform, higher pose accuracy is obtained predominantly at the cost of reduced frame rate and slightly increased, but still modest (up to approximately 10 W), power draw.

**Figure 5:**
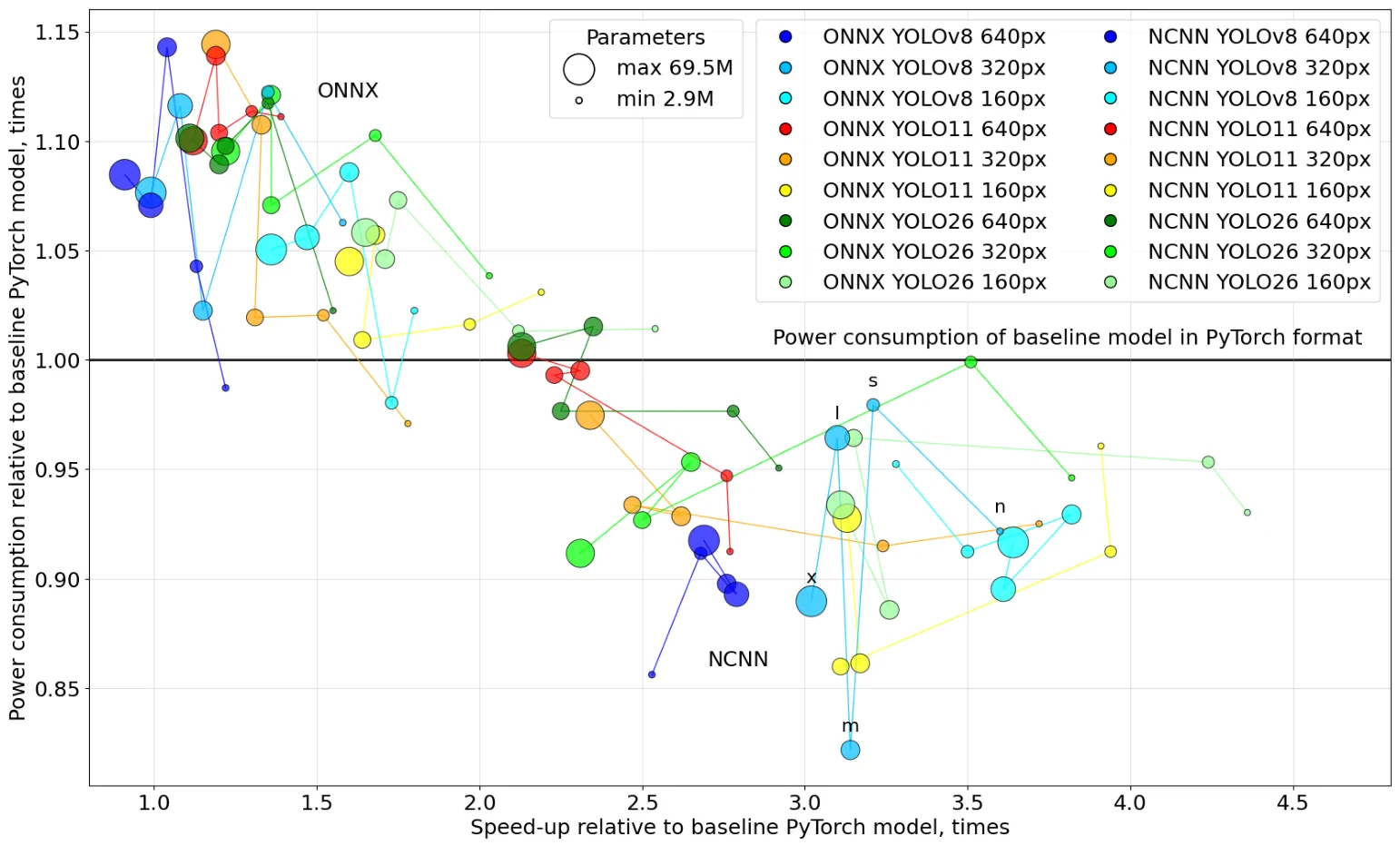
Power consumption and inference speed-up of ONNX and NCNN relative to the native PyTorch baseline, across YOLOv8-pose, YOLO11-pose, and YOLO26-pose models at three input resolutions (640×512 px, 320×256 px, and 160×128 px). Marker size denotes the number of model parameters (2.9 M–69.5 M).

Figure 5 compares the power consumption and inference speed-up of ONNX and NCNN relative to the native PyTorch baseline, with marker size denoting the number of model parameters (ranging from 2.9 M for the smallest nano variants to 69.5 M for the largest extra-large variants). ONNX consistently trades a modest increase in power draw, typically within about 15% of the PyTorch baseline (ratio ≈0.97–1.15×), for an inconsistent speed-up relative to PyTorch: for the smallest models ONNX still executes noticeably faster than PyTorch (up to approximately 2.6×), but this advantage narrows, and even reverses, for the largest, higher-parameter models at the highest input resolution, where ONNX latency can slightly exceed that of native PyTorch (speed-up as low as approximately 0.9×). NCNN, in contrast, achieves substantially higher and more consistent speed-ups across the full range of model scales. Contrary to the pattern observed for ONNX, NCNN’s speed-up advantage is not concentrated in the largest models: it is largest for the smallest models at reduced resolution (up to approximately 4.3×) and smaller, though still substantial, for the largest (*l*, *x*) models at the highest resolution (approximately 2.1–2.8×). NCNN keeps power consumption at or below the PyTorch baseline in nearly every configuration (ratio ≈0.83–1.01×), never exceeding it by a meaningful margin. This combination of consistently higher speed-up at power levels at or below the PyTorch baseline indicates that NCNN provides a markedly more favorable efficiency trade-off than ONNX for CPU-only edge deployment on the Raspberry Pi 5, once all three formats are run under the same fixed, matched multi-threaded CPU configuration.

### 4.4. Literature Context: Non-YOLO Architectures and Accelerator-Assisted Edge Systems

The model families evaluated in this study (YOLOv8-pose, YOLO11-pose, and YOLO26-pose) were chosen for consistency with our companion GPU-precision study [28] and because their single-stage, top-down design and mature ONNX/NCNN export tooling make them directly deployable in a CPU-only pipeline. Classic non-YOLO pose architectures: transformer-based ViTPose, attention-based RSN, and HRNet-family bottom-up detectors [19] have, to our knowledge, not been benchmarked in a CPU-only, accelerator-free deployment on a low-cost single-board computer; several of them (e.g., ViTPose) lack a mature, officially supported NCNN export path, and multi-branch high-resolution architectures such as HRNet are not designed for single-threaded CPU execution, making a like-for-like re-implementation on the Raspberry Pi 5 a substantial undertaking outside the scope of the present work.

To still situate our results relative to these architectures, Table 6 summarizes the best published accuracy and speed figures for representative non-YOLO pose estimators and for accelerator-assisted thermal pose-estimation systems from the literature discussed in Section 2. We stress that these figures were obtained by their respective authors on different thermal datasets, subject pools, and hardware, using their own training and evaluation protocols; they are reported here strictly as literature context and are *not* a controlled, same-dataset benchmark against our Raspberry Pi 5 results in Table 2.

Two observations follow. First, the transformer- and HRNet-based architectures achieve strong accuracy on thermal images when run on workstation-class GPUs, but none has been shown to run in an accelerator-free CPU pipeline; the lightweight, single-stage YOLO-pose design used here is, by contrast, exactly the class of architecture for which mature CPU-oriented export backends (ONNX Runtime, NCNN) exist. Second, none of the accelerator-assisted edge systems in Table 6 were evaluated CPU-only: THPoseLite depends on a Coral Edge TPU, and both OpenThermalPose2 and ThermiKit rely on GPU- or NPU-class accelerators substantially more powerful than the four-core Cortex-A76 CPU of the Raspberry Pi 5. This absence of any published CPU-only, accelerator-free thermal pose-estimation benchmark, across model families, scales, export formats, and resolutions, with matched latency, power, and energy measurement, is precisely the gap this study is designed to fill, and is why a same-dataset, same-hardware baseline could not be constructed from existing published systems.

### 4.5. Cross-Dataset Generalization and Multi-Person Scenes

The single-person thermal dataset used for training in this study is the same dataset previously collected and used in our earlier work evaluating embedded GPU deployment of thermal pose-estimation models on Jetson platforms [28]. To assess whether models trained on this dataset generalize beyond the single sensor and single subject it was collected from, the YOLO26s-pose model (640 × 512 px), trained exclusively on our thermal dataset, was evaluated in a strict zero-shot setting, with no fine-tuning of any kind, on the independent test splits of two publicly available thermal pose datasets: OpenThermalPose2 [22] and UCH-Thermal-Pose [19]. Table 7 reports box- and pose-level precision, recall, mAP50, and mAP50–95 for all three test splits under this protocol.

On our own held-out test split, the model achieves 0.984 box mAP50–95 and 0.942 pose mAP50–95, consistent with the YOLO26-s, 640 × 512 px result reported in Table 2. Applied zero-shot to OpenThermalPose2 and UCH-Thermal-Pose, pose mAP50–95 decreases to 0.617 and 0.544, respectively. Notably, box mAP50–95 degrades comparatively less across datasets (0.984, 0.856, 0.788) than pose mAP50–95, indicating that person localization generalizes reasonably well across sensors and imaging conditions, while precise keypoint placement is more strongly affected by the distribution shift. We attribute this discrepancy primarily to differences in the distribution of body poses and postures represented in each dataset: our dataset was collected under a comparatively narrower and more repetitive set of postures and activities, whereas OpenThermalPose2 and UCH-Thermal-Pose contain a broader and partly out-of-distribution range of body configurations, so that even correctly localized subjects can exhibit keypoint-placement mismatches when their pose falls outside the range represented during training.

**Figure 6:**
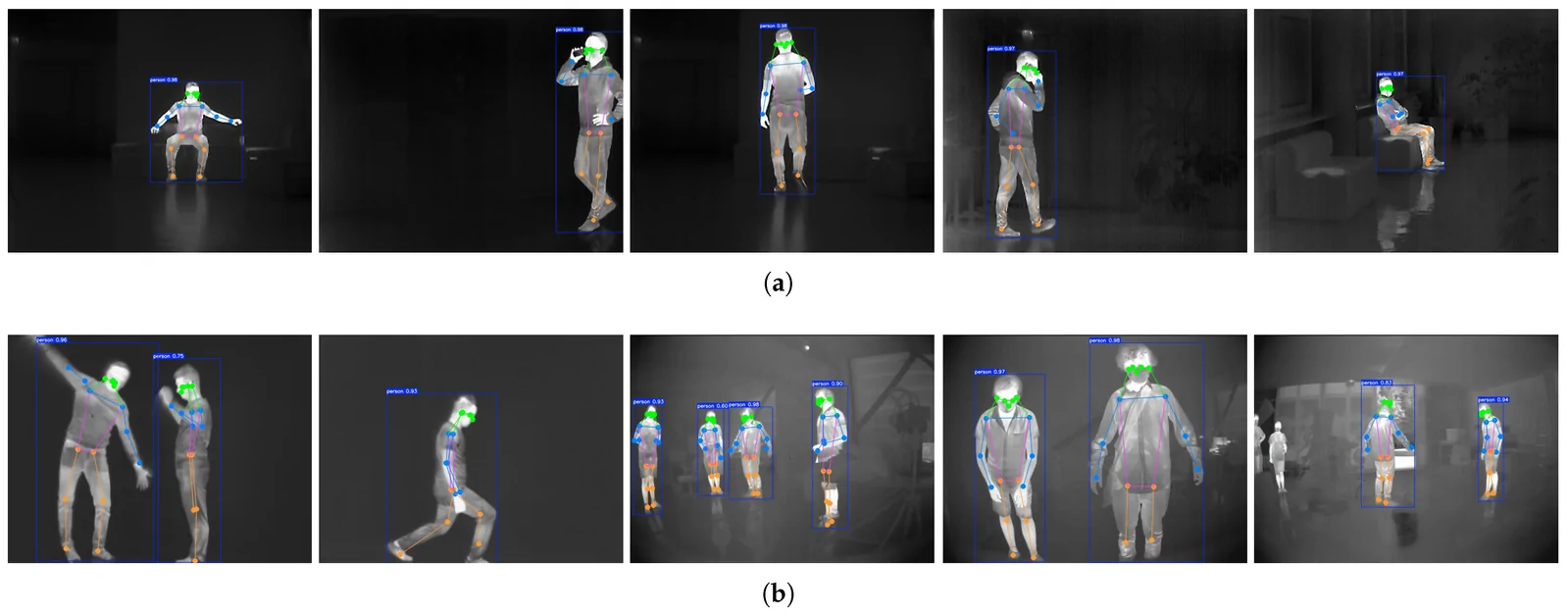
Qualitative predicted keypoints: (**a**) on our dataset (in-distribution), and (**b**) in a zero-shot setting on external thermal images from OpenThermalPose2 [22] and UCH-ThermalPose Set-B [19].

Figure 6 illustrates this qualitatively, showing predicted keypoints Figure 6a on our dataset and Figure 6b on external images from OpenThermalPose2 [22] and UCH-Thermal-Pose [19]. Several of the external example images in Figure 6b contain multiple occupants within the same frame, a scenario entirely absent from our single-person training data. The model, despite being trained and architecturally configured for single-person detection, produces separate, plausible bounding boxes and keypoint sets for each visible individual in these multi-occupant frames. This suggests that the underlying keypoint-localization capability generalizes qualitatively to multi-person scenes, even though it was neither trained nor quantitatively benchmarked for this setting. We emphasize that this observation is qualitative: Table 7 reports aggregate metrics per dataset rather than isolating multi-person accuracy, and a dedicated quantitative evaluation of multi-person performance (e.g., per-person mAP, identity assignment, and occlusion handling) remains necessary future work. Taken together, this cross-dataset, zero-shot, and qualitatively multi-person evaluation directly addresses the external-validation and multi-person generalization questions raised for the single-dataset, single-subject characterization reported elsewhere in this study.

We note that Figure 6b already provides qualitative failure-case evidence for this model: predicted keypoints on out-of-distribution postures and multi-occupant frames from OpenThermalPose2 and UCH-Thermal-Pose illustrate where keypoint placement degrades under distribution shift, consistent with the pose mAP50–95 drop reported in Table 7. A quantitative breakdown of accuracy by occlusion level, subject-to-camera distance, or pose type would require occlusion/pose-type annotations not present in the current dataset, and we identify this stratified analysis, together with per-keypoint AP reporting, as necessary future work rather than something addressable within the scope of the present revision.

## 5. Discussion

This study provides a unified evaluation of thermal pose estimation accuracy and CPU-only deployment efficiency by combining a previously annotated single-person thermal dataset with on-device latency, power, and energy measurements on a Raspberry Pi 5. The results show that, in contrast to GPU-accelerated platforms where numerical precision reduction is the primary efficiency driver, on CPU-only hardware the export format itself, rather than any change in numerical representation, is the dominant factor governing the achievable latency–power–energy trade-off (Table 2). This distinction is important for smart-home deployment, where the choice of computing platform determines not only which optimization strategies are available but also which trade-offs are relevant in practice [41,42].

For all three YOLO-pose generations examined, converting the native PyTorch models to ONNX or NCNN preserves pose accuracy almost exactly, with mean degradation confined to at most half a percentage point and standard deviations on the order of a few tenths of a percentage point across model scales (Table 4). This behavior differs markedly from the accuracy loss reported for INT8 quantization on embedded GPU platforms, where numerical precision reduction directly degrades keypoint localization [22]. Because ONNX and NCNN conversion on the Raspberry Pi 5 involves graph optimization and operator fusion rather than a reduction in numerical precision, the resulting models retain the full accuracy of the PyTorch baseline while substantially improving runtime efficiency. This finding indicates that, for CPU-only edge deployment of the lightweight, single-stage YOLO-pose architectures evaluated here, export-format conversion is a low-risk optimization with no meaningful accuracy–efficiency trade-off to negotiate within the scope of the models, dataset, and hardware tested; whether this holds for other pose-estimation architectures or more demographically and environmentally diverse thermal data remains to be verified.

Among the three formats, NCNN consistently provides the most favorable operating point. It achieves the lowest per-image latency and, consequently, the lowest energy consumption per frame of the three formats, despite not always drawing the lowest instantaneous power (Table 2, Figure 5). Native PyTorch and NCNN execution draw comparable power, with NCNN typically at or slightly below the PyTorch level, while ONNX Runtime consistently draws the highest power of the three formats through more aggressive multi-threaded CPU utilization, without a correspondingly larger, or even any, reduction in latency; for the largest models at the highest resolution, ONNX latency can match or slightly exceed that of native PyTorch. As a result, ONNX incurs higher energy consumption per frame than NCNN across essentially all configurations, and native PyTorch, despite comparable power draw to NCNN, incurs higher energy consumption because of its substantially longer execution time. This mirrors the broader principle that energy efficiency is determined by the joint effect of power draw and execution duration rather than by either quantity in isolation. These results are consistent with NCNN’s ARM NEON-optimized, packed-weight convolution kernels, which are purpose-built for mobile and embedded CPUs [27]; we note that this explanation follows from NCNN’s published design rationale together with our end-to-end latency and power measurements, rather than from operator-level profiling (e.g., perf-based instruction counts, cache-miss rates, or explicit NEON intrinsic call counts) performed in this study. Within the single-subject, single-sensor evaluation reported here, NCNN was consistently the most favorable of the three formats evaluated for real-time thermal pose estimation on the Raspberry Pi 5; broader validation across subjects, sensors, and deployment sites, together with operator-level profiling to establish the precise micro-architectural mechanism, would be needed before generalizing this recommendation further.

Beyond export format, input resolution and model scale define a consistent accuracy–latency–energy trade-off (Figure 3 and Figure 4). Larger models and higher resolutions improve pose mAP50–95, reaching up to 95.6% for YOLO26-x at 640×512 px, but this comes at the cost of substantially longer per-image time and higher energy consumption per frame. Notably, the accuracy gain from the largest (*x*) relative to the mid-sized (*m*) variants is comparatively modest at every resolution, while the corresponding increase in latency and energy is disproportionately large, indicating diminishing returns from scaling model capacity on CPU-only hardware. This pattern is consistent with prior evidence that keypoint localization in thermal imagery is fundamentally limited by sensing-induced degradation, such as coarse spatial detail and weak thermal contrast, rather than by model capacity alone [12,21,31], suggesting that beyond a moderate scale, further increases in model size primarily add computational cost without proportionally improving keypoint quality. From a deployment perspective, this supports selecting small or medium model variants at reduced resolution for applications that require only coarse posture or keypoint estimates, reserving larger models and higher resolutions for applications that specifically require fine-grained keypoint precision. We note that the present study evaluates pose-estimation accuracy and deployment efficiency only; translating these operating points into concrete downstream tasks such as activity recognition or fall detection would require dedicated, task-specific evaluation, which we identify as future work.

Compared with embedded GPU platforms, the absolute latency achieved on the Raspberry Pi 5 remains substantially higher. Prior work converting YOLO11-pose to TensorRT FP16 on a Jetson AGX Orin reports latencies as low as a few milliseconds per frame [22], whereas the fastest NCNN configuration on the Raspberry Pi 5 requires 8–9 ms per frame only for the smallest models at the lowest resolution, with larger models and higher resolutions extending latency into the hundreds or low thousands of milliseconds. This gap reflects the absence of any dedicated neural-network accelerator on the Raspberry Pi 5, in contrast to both Jetson-class GPUs and USB-attached accelerators such as the Coral Edge TPU, which has been used to reach real-time thermal pose inference within a 6 W budget [21]. Nevertheless, the Raspberry Pi 5 achieves frame rates well above real-time, exceeding 100 FPS, for nano-scale models at 160×128 px, and sustains power consumption at or below approximately 10 W for the NCNN configurations recommended for deployment (Table 2), noting that ONNX Runtime alone draws marginally more, up to 11.4 W, for the largest models at the highest resolution, indicating that, for pose-estimation applications that do not require the highest keypoint precision, CPU-only inference on low-cost, widely available hardware remains a practical alternative to GPU- or accelerator-based deployment. This conclusion pertains to pose-estimation throughput, accuracy, and power consumption; it does not by itself establish suitability for any specific downstream activity-recognition task, which was not evaluated in this study. This is consistent with related evidence that Raspberry Pi-class devices, when paired with appropriately optimized models, can sustain real-time, privacy-aware visual analytics for smart-home and building-monitoring applications [24,25,26].

The present study did not explore quantization-based optimization, such as 8-bit integer inference, which on the Raspberry Pi 5 would need to rely on CPU-native quantization schemes rather than the Tensor Core-accelerated INT8 execution available on Jetson platforms. Given that quantization on GPU-class hardware has been shown to introduce configuration-dependent accuracy degradation [22], and that CPU-only quantization kernels are typically less mature than their floating-point counterparts, the accuracy–efficiency benefit of quantization on CPU-only edge devices remains an open question. Future work could investigate CPU-targeted quantization, structured pruning, or knowledge distillation into more compact backbones as complementary strategies for further reducing latency and energy consumption beyond what export-format conversion alone provides, particularly for the larger model scales where the accuracy gains observed in this study were disproportionately small relative to their computational cost.

This study evaluated a thermal dataset acquired from a single adult participant with one thermal sensor, recorded across eight indoor rooms at a fixed ambient temperature (21 °C) and under three clothing conditions. While this setup introduces some environmental and appearance variability, the use of a single participant limits the demographic and morphological diversity (e.g., age, gender, body shape) represented in the training and test data, and the reported accuracy should be interpreted as characterizing this participant and sensor rather than a population-level estimate. While the train/validation/test split was performed at the recording-session level to prevent temporally adjacent frames from leaking across partitions, all sessions were recorded with the same participant and camera, so the test set remains a within-dataset, same-subject split rather than an independent, cross-scene, or cross-subject evaluation. Generalization to other individuals, multi-person scenes, different room layouts, or sensors with different noise and contrast characteristics remains to be established [30]. Future work should extend the dataset to multiple participants spanning a wider range of ages, body shapes, and clothing to validate these findings at a population level. We also note that Section 4.2 and Table 3 report within-run measurement variability and on-chip temperature/thermal-throttling status for all 135 configurations, which rules out thermal throttling as a confound in the reported latency and power figures; however, this does not constitute the operator-level profiling (CPU utilization breakdown, cache/memory behavior, NEON instruction evidence) that would be needed to establish the precise micro-architectural mechanism behind NCNN’s efficiency advantage, and we identify such profiling as necessary future work. In addition, the benchmarking protocol executed each model configuration as an independent, sequential run. We monitored on-chip CPU temperature and the firmware thermal-throttling flag throughout every run (Table 3, Section 4.2) and found no evidence of throttling in any of the 135 configurations, with the highest instantaneous temperature reaching 77.4 °C under fan cooling at 21 °C ambient; however, this does not rule out throttling under longer, back-to-back continuous operation spanning many sequential runs, which was not evaluated. Similarly, because each configuration was executed only once, the reported power and latency values reflect a single measurement run per configuration; we report the within-run standard deviation of these measurements in Table 3, but the between-run (independent-repetition) variance has not been characterized. The reported accuracy–latency–power trade-offs should therefore be interpreted as deployment-oriented evidence under controlled, short-duration residential conditions rather than as a claim of broad domain or long-term operational generalization.

## 6. Conclusions

This study investigated efficient thermal pose estimation, a key building block for privacy-preserving smart-home activity-recognition pipelines, on a CPU-only edge platform, combining a previously annotated single-person thermal dataset with deployment of three successive YOLO-pose generations on a Raspberry Pi 5 single-board computer. A comprehensive evaluation was conducted to assess the accuracy, latency, power, and energy trade-offs of YOLOv8-pose, YOLO11-pose, and YOLO26-pose models across five model scales, three export formats, and three input resolutions. The findings indicate that unlike GPU-accelerated edge platforms where numerical-precision reduction is the primary efficiency driver, export-format conversion is the dominant factor governing runtime efficiency on CPU-only hardware.

Among the three formats examined, and within the scope of the single-subject, single-thermal-sensor dataset used in this study, NCNN was identified as the most favorable option for edge deployment. NCNN preserves pose accuracy nearly identically to native PyTorch (v2.10.0) execution, while achieving substantial reductions in per-image latency and, consequently, the lowest energy consumption per frame across all evaluated model families and resolutions. ONNX also preserves accuracy, but its power draw is consistently the highest of the three formats while its speed-up over PyTorch is inconsistent, ranging from a modest improvement for smaller models to little or no benefit, and occasionally a slight slowdown, for the largest models at the highest resolution, making it a less favorable choice than NCNN for continuous residential operation. Input resolution and model scale define a consistent accuracy–efficiency trade-off: higher resolutions and larger models improve pose mAP50–95, reaching up to 95.6% for YOLO26-x at 640×512 px, but with diminishing accuracy returns for the largest model scales relative to the substantial increase in latency and energy they incur. In addition, absolute latency on the Raspberry Pi 5 remains an order of magnitude higher than that reported for GPU-accelerated Jetson platforms, reflecting the absence of a dedicated neural-network accelerator; nevertheless, nano-scale models at reduced resolution sustain frame rates well above real-time (exceeding 100 FPS) at power levels below 10 W, confirming the viability of low-cost, CPU-only hardware for real-time thermal pose estimation, which is a prerequisite for smart-home activity-recognition applications but was not itself evaluated as a downstream task in this study.

Future research could investigate CPU-targeted quantization, structured pruning, or knowledge distillation into more compact backbones to further reduce latency and energy consumption beyond what export-format conversion alone provides. Although the qualitative cross-dataset examples in Figure 6b suggest the model can produce plausible keypoint predictions for multiple occupants within a single frame, extending the evaluation to a dedicated, quantitatively benchmarked multi-person setting, additional thermal sensors, and sustained continuous operation would help assess the generalization and thermal-throttling robustness of the proposed deployment pipeline under realistic long-term residential conditions. In addition, evaluating the proposed pose-estimation pipeline within concrete downstream tasks, such as fall detection, long-lie detection, or activity classification, would be necessary to directly validate the smart-home application scenarios that motivate this work.

## Figures and Tables

**Table 1:** Model parameters and FLOPs across the three evaluated input resolutions for each YOLO-pose family and scale. GFLOPs are as reported by Ultralytics at model instantiation; opset/simplify/dynamic settings are as declared in Section 3.

Family	Scale	Params (M)	GFLOPs			Opset	Simplify	Dynamic
			640 × 512	320 × 256	160 × 128			
YOLOv8-pose	n	3.3	9.3	≈2.3	≈0.6	20	True	False
	s	11.6	30.4	≈7.6	≈1.9	20	True	False
	m	26.5	81.4	≈20.4	≈5.1	20	True	False
	l	44.5	169.2	≈42.3	≈10.6	20	True	False
	x	69.5	264.0	≈66.0	≈16.5	20	True	False
YOLO11-pose	n	2.9	7.5	≈1.9	≈0.5	20	True	False
	s	9.9	23.3	≈5.8	≈1.5	20	True	False
	m	20.9	71.9	≈18.0	≈4.5	20	True	False
	l	26.2	91.0	≈22.8	≈5.7	20	True	False
	x	58.8	203.8	≈51.0	≈12.7	20	True	False
YOLO26-pose	n	3.7	10.3	≈2.6	≈0.6	20	True	False
	s	11.8	29.2	≈7.3	≈1.8	20	True	False
	m	24.2	85.2	≈21.3	≈5.3	20	True	False
	l	28.6	103.6	≈25.9	≈6.5	20	True	False
	x	62.7	225.3	≈56.3	≈14.1	20	True	False

**Table 2:** Summary of per-image inference time, power consumption, energy per frame, and pose accuracy (mAP50–95) for YOLOv8-pose, YOLO11-pose, and YOLO26-pose models on the Raspberry Pi 5, across three export formats (native PyTorch, ONNX, NCNN) and three input resolutions. Per-image time includes preprocessing, inference, and postprocessing. PT denotes PyTorch. mAP50–95 is reported for the native PyTorch model; format-wise degradation is quantified separately.

Model		Input Resolution	Time/Frame, ms			Power, W			Energy/Frame, J			mAP50–95, %
			PT	ONNX	NCNN	PT	ONNX	NCNN	PT	ONNX	NCNN	
YOLOv8-pose	n	640×512	209	172	83	8.6	8.5	7.4	1.80	1.46	0.61	90.3
	s		483	427	180	9.4	9.8	8.6	4.55	4.20	1.55	93.4
	m		1021	985	371	9.5	10.9	8.5	9.72	10.72	3.17	93.7
	l		1938	1954	695	10.3	11.0	9.2	19.93	21.53	6.39	94.1
	x		2712	2976	1007	10.5	11.4	9.6	28.42	33.84	9.68	94.2
	n	320×256	71	45	20	7.1	7.6	6.6	0.51	0.34	0.13	87.0
	s		144	107	45	7.9	8.8	7.7	1.13	0.94	0.35	89.1
	m		280	244	89	9.3	9.5	7.7	2.61	2.32	0.68	90.4
	l		513	475	166	9.3	10.3	8.9	4.75	4.90	1.48	93.6
	x		711	718	236	9.9	10.7	8.8	7.05	7.66	2.08	93.8
	n	160×128	28	16	9	6.7	6.8	6.4	0.19	0.11	0.05	75.0
	s		63	37	18	7.3	7.2	6.7	0.46	0.26	0.12	81.4
	m		123	77	32	7.4	8.0	6.9	0.91	0.62	0.22	84.6
	l		208	142	58	8.2	8.7	7.3	1.71	1.23	0.42	87.5
	x		285	209	78	8.4	8.8	7.7	2.40	1.85	0.60	88.6
YOLO11-pose	n	640×512	231	166	83	8.1	9.0	7.4	1.88	1.50	0.62	89.8
	s		500	386	182	8.9	9.9	8.4	4.43	3.81	1.52	90.2
	m		1237	1031	554	9.5	10.5	9.4	11.74	10.80	5.22	92.4
	l		1535	1293	665	9.4	10.7	9.3	14.40	13.81	6.21	93.0
	x		2916	2601	1370	9.9	10.9	9.9	28.77	28.23	13.56	93.6
	n	320×256	75	42	20	7.5	7.3	6.9	0.56	0.30	0.14	86.6
	s		145	95	45	8.0	8.2	7.3	1.16	0.78	0.33	88.4
	m		323	246	131	9.2	9.3	8.5	2.95	2.30	1.12	92.0
	l		406	306	155	8.9	9.9	8.3	3.62	3.02	1.28	92.0
	x		742	622	317	9.3	10.6	9.0	6.87	6.58	2.86	92.5
	n	160×128	31	14	8	6.7	6.9	6.4	0.21	0.10	0.05	73.8
	s		60	31	15	7.3	7.4	6.6	0.44	0.23	0.10	84.0
	m		118	72	38	7.6	7.7	6.6	0.90	0.55	0.25	84.3
	l		150	89	47	7.9	8.3	6.8	1.19	0.75	0.32	87.2
	x		279	174	89	8.4	8.7	7.8	2.33	1.52	0.69	88.9
YOLO26-pose	n	640×512	239	155	82	8.3	8.5	7.9	1.98	1.31	0.65	93.1
	s		521	387	187	8.8	9.8	8.6	4.58	3.80	1.61	94.2
	m		1261	1032	560	9.4	10.4	9.2	11.92	10.70	5.17	94.4
	l		1553	1292	660	9.6	10.4	9.7	14.86	13.46	6.41	95.2
	x		2876	2581	1350	9.9	10.9	10.0	28.48	28.01	13.45	95.6
	n	320×256	76	38	20	7.2	7.5	6.7	0.55	0.28	0.13	91.6
	s		157	93	45	7.6	8.4	7.6	1.19	0.78	0.34	92.0
	m		331	244	133	8.9	9.6	8.3	2.96	2.33	1.10	92.1
	l		411	303	155	8.7	9.7	8.3	3.57	2.95	1.28	93.5
	x		741	609	322	9.4	10.3	8.6	7.00	6.30	2.77	93.9
	n	160×128	34	13	8	6.9	7.0	6.4	0.23	0.09	0.05	81.1
	s		65	30	15	7.2	7.3	6.9	0.47	0.22	0.10	87.5
	m		120	69	38	7.4	8.0	7.2	0.89	0.55	0.27	87.5
	l		150	88	46	7.9	8.2	7.0	1.18	0.72	0.32	88.8
	x		279	169	90	8.2	8.7	7.7	2.30	1.47	0.69	89.3

**Table 3:** Within-run measurement variability and thermal state, aggregated across export format and input resolution (15 configurations per cell: three model families × five model scales). CV denotes the coefficient of variation (SD/mean × 100) computed from the 500 per-image observations within a single run; the bracketed range gives the minimum and maximum CV observed among the 15 configurations in that cell. Throttled runs indicates the number of configurations (out of 15) in which the vcgencmd get_throttled flag was nonzero at any point during the run.

Format	Resolution	Time CV, %	Power CV, %	Mean CPU Temp, °C	Max CPU Temp, °C	Throttled Runs
PT	640 × 512	0.8 [0.3–3.0]	7.6 [5.3–8.8]	67.7	76.3	0/15
PT	320 × 256	1.3 [0.5–2.5]	8.1 [6.2–9.6]	65.5	75.7	0/15
PT	160 × 128	1.9 [0.9–3.6]	6.8 [4.7–8.9]	62.5	68.1	0/15
ONNX	640 × 512	0.8 [0.1–2.5]	6.7 [4.7–9.3]	70.0	77.4	0/15
ONNX	320 × 256	2.0 [0.3–6.1]	8.9 [7.5–10.7]	66.9	77.4	0/15
ONNX	160 × 128	3.8 [0.5–12.7]	9.7 [7.1–10.9]	62.6	71.4	0/15
NCNN	640 × 512	1.9 [0.8–6.2]	8.1 [4.7–10.0]	66.0	74.7	0/15
NCNN	320 × 256	4.8 [1.3–12.1]	9.0 [5.2–10.9]	63.8	71.9	0/15
NCNN	160 × 128	2.7 [1.1–8.5]	6.7 [2.3–10.5]	59.8	66.4	0/15

**Table 4:** Mean pose mAP50–95 degradation of ONNX and NCNN relative to native PyTorch, reported in percentage points (pp) as mean ± standard deviation, averaged over five model scales (*n*–*x*) for YOLOv8-pose, YOLO11-pose, and YOLO26-pose at each input resolution.

Export Format	640×512 pix	320×256 pix	160×128 pix
ONNX	−0.54±0.31 pp	−0.2±0.13 pp	0.04±0.31 pp
NCNN	−0.52±0.28 pp	−0.2±0.13 pp	0.02±0.24 pp

**Table 5:** Pareto-optimal configurations (NCNN format) in the accuracy–latency plane, derived from Table 2. A configuration is Pareto-optimal if no other of the 45 evaluated NCNN configurations (three model families × five scales × three resolutions) achieves both lower latency and equal-or-higher pose mAP50–95.

Model	Resolution	Time/Frame, ms (NCNN)	mAP50–95, %
YOLO26-n	160 × 128	8	81.1
YOLO26-s	160 × 128	15	87.5
YOLO26-n	320 × 256	20	91.6
YOLO26-s	320 × 256	45	92.0
YOLO26-n	640 × 512	82	93.1
YOLO26-l	320 × 256	155	93.5
YOLOv8-l	320 × 256	166	93.6
YOLO26-s	640 × 512	187	94.2
YOLO26-m	640 × 512	560	94.4
YOLO26-l	640 × 512	660	95.2
YOLO26-x	640 × 512	1350	95.6

**Table 6:** Literature-reported pose-estimation accuracy and inference speed for representative non-YOLO architectures and accelerator-assisted thermal edge-deployment systems, compared with our best Raspberry Pi 5 CPU-only (NCNN) result. Datasets, subjects, and hardware differ across rows; values are not directly comparable and are included for context only.

Method	Architecture family	Dataset	Hardware	Format	Pose AP/mAP	Speed
ViTPose [19]	Vision Transformer (top-down)	UCH-Thermal-Pose	Tesla V100 GPU	PyTorch	AP =0.915	15.3 FPS
RSN [19]	CNN + attention (top-down)	UCH-Thermal-Pose	Tesla V100 GPU	PyTorch	AP =0.890	15.4 FPS
CenterNet (HRNet-W32) [19]	HRNet backbone (bottom-up)	UCH-Thermal-Pose	Tesla V100 GPU	PyTorch	AP =0.795	12.5 FPS
Bottom-Up HRNet [19]	HRNet backbone (bottom-up)	UCH-Thermal-Pose	Tesla V100 GPU	PyTorch	AP =0.662	1.5 FPS
OpenPose [19]	VGG-19, PAF (bottom-up)	UCH-Thermal-Pose	Tesla V100 GPU	PyTorch	AP =0.806	13.0 FPS
THPoseLite [21]	MobileNetV2 regression (single-person)	In-house Almeria dataset	Raspberry Pi 4 + Coral Edge TPU	INT8	MSE =35.48 ^a^	12.28 FPS (<6 W)
YOLO11n-pose [22]	YOLO (top-down CNN)	OpenThermal-Pose2	Jetson AGX Orin	TensorRT FP16	${\mathrm{AP}}_{50:95}\approx 0.85$	≈3.2 ms/frame
YOLOv11n-pose [23]	YOLO (top-down CNN)	In-house multi-camera (Thermal1)	Sub-1 TOPS edge NPU	Vendor SDK	${\mathrm{mAP}}_{50:95}=0.7245$	Real-time (unspecified)
Ours: YOLO26n, 640 × 512	YOLO (top-down CNN)	Our thermal dataset	Raspberry Pi 5 (CPU-only)	NCNN	${\mathrm{mAP}}_{50:95}=0.931$	12.2 FPS (7.9 W)

^a^ THPoseLite is evaluated with mean-squared landmark error (MSE) rather than COCO-style OKS-based AP, and performs single-person 22-point regression rather than detection-plus-pose estimation; its metric is therefore not numerically comparable to the AP/mAP values reported for the other systems.

**Table 7:** Zero-shot cross-dataset validation of the YOLO26s-pose model (640×512 px), trained exclusively on our thermal dataset, evaluated without fine-tuning on the test splits of our dataset, OpenThermalPose2 [22], and UCH-ThermalPose Set-B [19].

Dataset	Box				Pose			
	P	R	mAP50	mAP50–95	P	R	mAP50	mAP50–95
Ours [28]	0.996	0.992	0.995	0.984	0.996	0.992	0.995	0.942
OpenThermalPose2 [22]	0.992	0.962	0.986	0.856	0.906	0.871	0.904	0.617
UCH-Thermal-Pose [19]	0.975	0.719	0.85	0.788	0.941	0.694	0.825	0.544

## Data Availability

The original data presented in this study, and the supplementary scripts are openly available in Mendeley at https://data.mendeley.com/datasets/64dhsznpfx/3 (accessed on 30 May 2026).
